# Prevalence and outcomes of malaria as co-infection among patients with human African trypanosomiasis: a systematic review and meta-analysis

**DOI:** 10.1038/s41598-021-03295-8

**Published:** 2021-12-10

**Authors:** Kwuntida Uthaisar Kotepui, Frederick Ramirez Masangkay, Giovanni De Jesus Milanez, Manas Kotepui

**Affiliations:** 1 Medical Technology, School of Allied Health Sciences, Walailak University, Tha Sala, Nakhon Si Thammarat, Thailand; 2 Department of Medical Technology, Institute of Arts and Sciences, Far Eastern University-Manila, Manila, Philippines; 3 Department of Medical Technology, Faculty of Pharmacy, Royal and Pontifical University of Santo Tomas, Manila, Philippines

**Keywords:** Malaria, Parasitic infection

## Abstract

Human African trypanosomiasis (HAT) is endemic in Africa; hence, the possibility of co-infection with malaria among patients with HAT exists. The present study investigated co-infection with malaria among patients with HAT to provide current evidence and characteristics to support further studies. Potentially relevant studies that reported *Plasmodium* spp. infection in patients with HAT was searched in PubMed, Web of Science, and Scopus. The risk of bias among the included studies was assessed using the checklist for analytical cross-sectional studies developed by the Joanna Briggs Institute. The pooled prevalence of *Plasmodium* spp. infection in patients with HAT was quantitatively synthesized using a random-effects model. Subgroup analyses of study sites and stages of HAT were performed to identify heterogeneity regarding prevalence among the included studies. The heterogeneity of the outcome among the included studies was assessed using Cochran’s Q and *I*^*2*^ statistics for consistency. Publication bias was assessed if the number of included studies was 10 or more. For qualitative synthesis, a narrative synthesis of the impact of *Plasmodium* spp. infection on the clinical and outcome characteristics of HAT was performed when the included studies provided qualitative data. Among 327 studies identified from three databases, nine studies were included in the systematic review and meta-analysis. The prevalence of *Plasmodium* spp. co-infection (692 cases) among patients with HAT (1523 cases) was 50% (95% confidence interval [CI] = 28–72%, *I*^2^ = 98.1%, seven studies). Subgroup analysis by type of HAT (gambiense or rhodesiense HAT) revealed that among patients with gambiense HAT, the pooled prevalence of *Plasmodium* spp. infection was 46% (95% CI = 14–78%, *I*^2^ = 96.62%, four studies), whereas that among patients with rhodesiense HAT was 44% (95% CI = 40–49%, *I*^2^ = 98.3%, three studies). Qualitative syntheses demonstrated that *Plasmodium* spp. infection in individuals with HAT might influence the risk of encephalopathy syndrome, drug toxicity, and significantly longer corrected QT time. Moreover, longer hospital stays and higher treatment costs were recorded among co-infected individuals. Because of the high prevalence of malaria among patients with HAT, some patients were positive for malaria parasites despite being asymptomatic. Therefore, it is suggested to test every patient with HAT for malaria before HAT treatment. If malaria is present, then antimalarial treatment is recommended before HAT treatment. Antimalarial treatment in patients with HAT might decrease the probability of poor clinical outcomes and case fatality in HAT.

## Introduction

Human African trypanosomiasis (HAT), or sleeping sickness, is a fatal disease endemic in sub-Saharan Africa, and 10 million people reside in areas at risk^1^. The most recent study revealed that 977 cases of HAT were reported in 2018, which was down from 2164 in 2016^2^. HAT is caused by the protozoa *Trypanosoma* spp. and transmitted via the tsetse fly (*Glossina* spp.)^1^. The two variants of HAT have been reported, and they are caused by either *Trypanosoma brucei gambiense* (*T. b. gambiense*) or *Trypanosoma brucei rhodesiense* (*T. b. rhodesiense*). Between these species, *T. b. gambiense* accounts for more than 95% of all cases of HAT^3^. *T. b. gambiense* is endemic to West Africa, whereas *T. b. rhodesiense* is endemic to East Africa^4^. The reservoir hosts for *T. b. gambiense* are humans, whereas the hosts for *T. b. rhodesiense* are domestic and wild animals^5^. HAT is classified into early and late stages. After an initial bite by the tsetse fly, parasites enter the hemolymphatic system in the early stage and then multiply and spread through the bloodstream, lymphatic system, and systemic organs^4^. In the late stage, parasites cross the blood–brain barrier into the CNS, causing various neurological signs and symptoms such as sleep disorder with daytime somnolence and nocturnal insomnia (i.e., sleeping sickness)^6^, and other symptoms including slurred speech, cerebellar ataxia, tremors, headache, myositis, and myelopathy^7^. If patients are left untreated, the disease deteriorates progressively into impaired consciousness, incontinence, seizures, and eventually death in most cases^3^.

Malaria is a major cause of death among children younger than 5 years in Africa, where malaria is highly endemic^8^. WHO reported an estimated 229 million cases of malaria and 409,000 deaths in 2019^9^. Malaria is caused by one of the six *Plasmodium* species (*P. falciparum*, *P. vivax*, *P. malariae*, *P. ovale curtisi*, *P. ovale wallikeri*, and *P. knowlesi*), which are transmitted via the bite of *Anopheles* mosquitoes harboring parasites^10^. Signs and symptoms of malaria range from asymptomatic to severe complications depending on the causative *Plasmodium* species, parasitemia level, and immune status of the patient^11–15^. Severe *P. falciparum* malaria as defined by the most recent WHO criteria by signs including impaired consciousness, prostration, multiple convulsions, acidosis, hypoglycemia, severe malarial anemia, renal impairment, jaundice, pulmonary edema, significant bleeding, shock, and hyperparasitemia^16^. *P. falciparum* and *P. knowlesi* are the main causes of severe malaria and death, whereas other *Plasmodium* species less frequently cause severe malaria^14,15,17^.

As both HAT and malaria are endemic to Africa, the possibility of co-infection by these two diseases cannot be discounted. Therefore, the present study investigated current evidence regarding malaria and HAT co-infection in the literature via a systematic review and meta-analysis. In addition, the characteristics of co-infected patients were qualitatively and quantitatively synthesized to provide evidence-based information for further studies.

## Methods

### Protocol and registration

The systematic review and meta-analysis protocol was registered at PROSPERO (ID: CRD42021266397) and followed the Preferred Reporting Items for Systematic Reviews and Meta-Analyses statement^18^.

### Search strategy

The search strategy involved the use of combinations of the following search terms: “(Malaria OR Plasmodium) AND (Trypanosomiases OR “Sleeping Sickness” OR “African Sleeping” OR Nagana OR Trypanosome OR Nannomona) AND (co-infection OR co-infection OR “Co-infection” OR mixed OR concurrent OR Polymicrobial OR multiple OR dual)” (Table S1). All relevant search terms were retrieved from the Medical Subject Heading in The National Center for Biotechnology Information. The searches were performed in PubMed, Web of Science, and Scopus on July 4, 2021, without restriction on language or publication year. Additional searches of the reference lists of the included studies and Google Scholar were performed to assure that all relevant studies were captured in the search protocol.

### Eligibility criteria

The eligibility criteria were related to participants (P), phenomena of interest (I), and context (Co) or PICo. The primary outcome of this study was the pooled prevalence of *Plasmodium* spp. infection among patients with HAT globally. Therefore, P was patients with HAT, I was *Plasmodium* spp. infection and Co was worldwide context. Original studies (retrospective or prospective observational, cross-sectional studies, cohort, clinical trials, or case–control studies) that reported *Plasmodium* spp. infection among patients with HAT was included. Conversely, case studies, case series, letters to the editors, commentary, reviews, systematic review, in vitro studies, and in vivo studies were excluded.

### Study selection

Study selection was performed independently by two authors (KUK, MK). Discrepancies between the authors were resolved through discussion until a consensus was reached. After duplicates were removed, the remaining studies were screened for titles and abstracts. After non-relevant studies were excluded, the full text of the remaining studies was examined according to the eligibility criteria. Then, studies that did not meet the eligibility criteria were excluded for specific reasons. Finally, studies that met the eligibility criteria were included for further data extraction.

### Data extraction

Studies were extracted into a standardized pilot datasheet under the following categories: name of the first author, year of publication, study site, the year when the study was conducted, study design, number of participants enrolled, characteristics of participants, age, male ratio, number of infecting *Plasmodium* spp. among patients with HAT, number of patients with HAT, malaria diagnostic method, and HAT diagnostic method.

### Risk of bias

The risk of bias among the included studies was assessed using the checklist for analytical cross-sectional studies developed by the Joanna Briggs Institute (JBI)^19^. The JBI tool assessed eight characteristics of the included studies: inclusion criteria of the participants, study subjects and setting, measurement of exposure, measurement of the condition, identification of the confounding factor, measurement of outcome, and appropriateness of statistical analysis. Scores of 7–8, 4–6, and < 4 indicated a low, moderate, and high risk of bias, respectively.

### Data syntheses

Data syntheses included qualitative and quantitative syntheses. For quantitative synthesis, statistical analysis was used to determine the pooled prevalence of *Plasmodium* spp. infection among patients with HAT. Subgroup analyses were performed by types of HAT (gambiense or rhodesiense HAT) to identify the source of heterogeneity regarding prevalence among the included studies. The heterogeneity of the outcome among the included studies was assessed using Cochran’s Q and the *I*^2^ statistic for consistency. Cochran’s Q less than 0.05 or *I*^2^ greater than 50% indicated substantial heterogeneity of the outcome among the included studies. Pooled analysis of the prevalence was performed using a random-effects model^20^. Publication bias was assessed if the number of the included studies was at least 10^21^. All quantitative analyses were performed using Stata version 14 (StataCorp, College Station, TX, USA). For qualitative synthesis, a narrative synthesis of the impact of *Plasmodium* spp. infection on clinical and outcome characteristics of patients with HAT was performed when the included studies reported qualitative data.

## Results

### Search results

**Figure 1:**
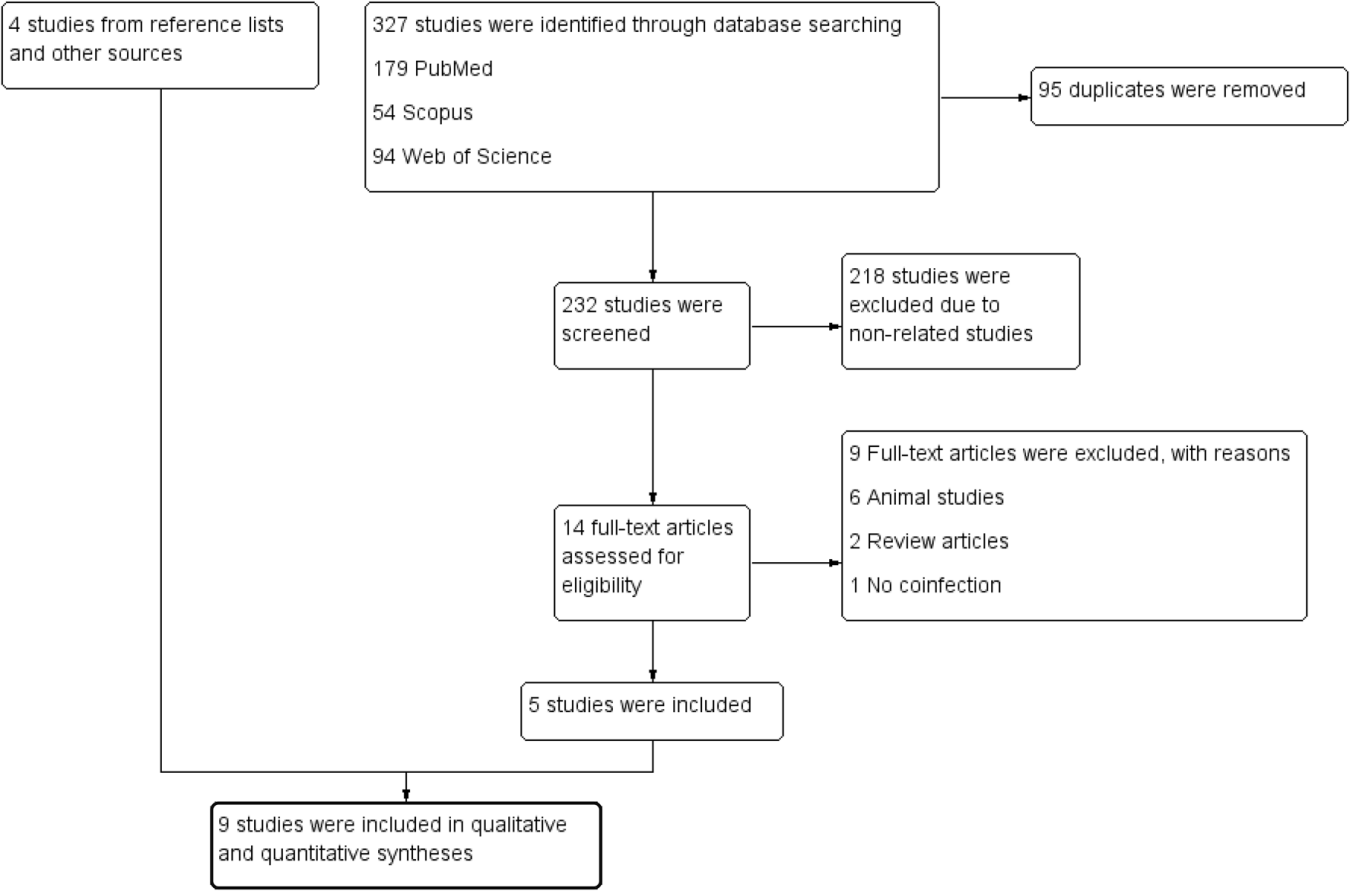
Study flow diagram demonstrated the selection of relevant studies.

Three hundred twenty-seven studies were identified in PubMed (179 studies), Web of Science (94 studies), and Scopus (54 studies). After 95 duplicates were removed, 232 studies were screened for titles and abstracts. After 218 non-relevant studies were excluded, the full text of 14 studies was examined. After screening the full text of the studies, six animal studies, two review articles, and one article in which no patients with HAT had malaria were excluded. Five studies^22–26^ were included in the systematic review. An additional four studies^6,27–29^ were identified from searches of reference lists. Finally, nine studies^6,22–29^ were included in the systematic review and meta-analysis (Fig. 1).

### Characteristics of the included studies

**Figure 2:**
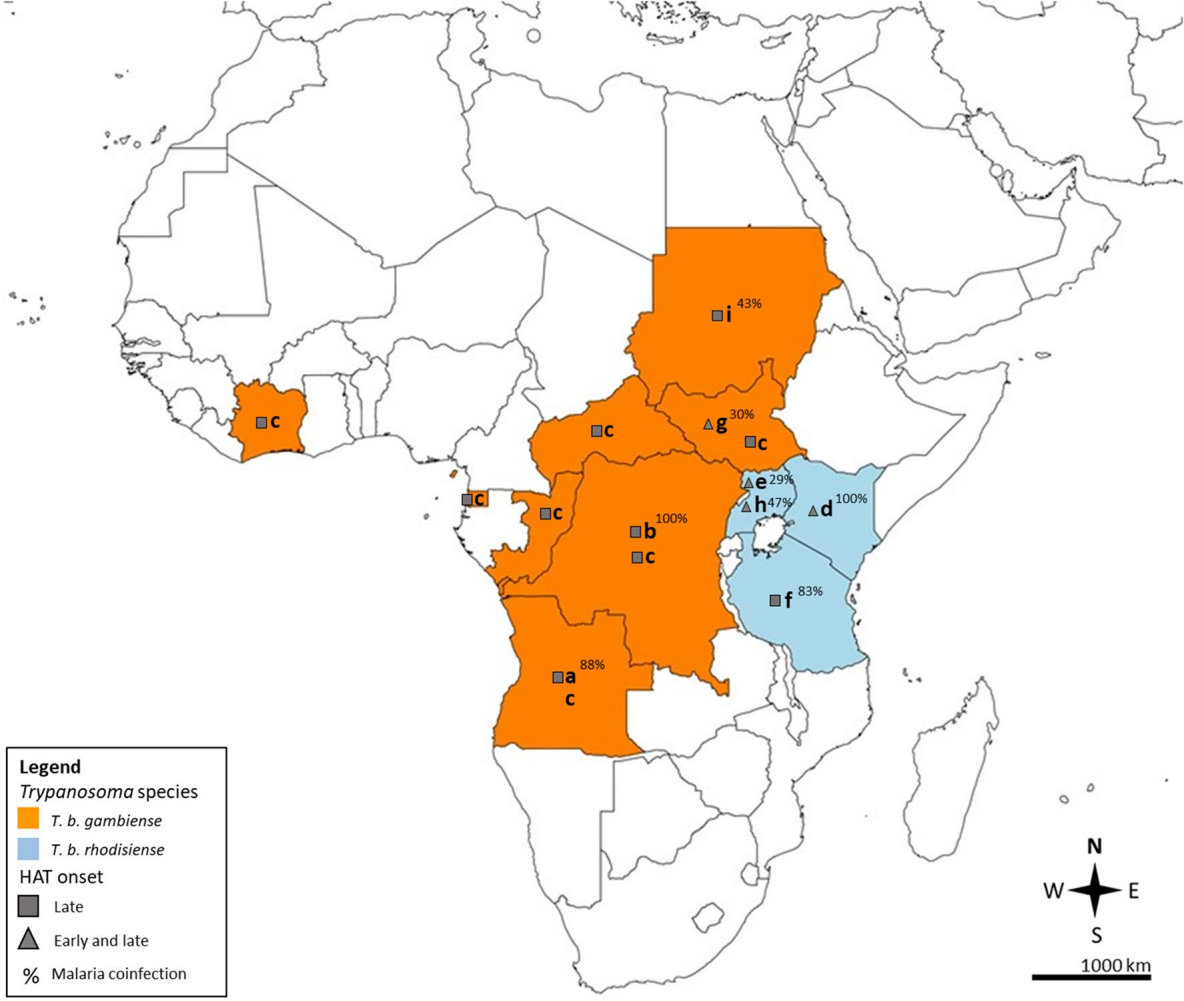
Epidemiology of Human African trypanosomiasis (HAT) and malaria co-infection in the African continent. *HAT* human African trypanosomiasis; *T. b. Trypanosoma brucei*; (**a)** Angola; (**b)** Democratic Republic of Congo; (**c)** Angola, Central African Republic, Cˆote d’Ivoire, Democratic Republic of Congo, Republic of Congo, Equatorial Guinea, and South Sudan; (**d)** Kenya; (**e)** Uganda; (**f)** Tanzania and Uganda; (**g)** South Sudan; (**h)** Uganda; (**i)** Sudan. Map was retrieved and modified by the authors from https://mapchart.net/world.html. Authors are allowed to use, edit, and modify any map created with mapchart.net for publication freely by adding the reference to mapchart.net.

All included studies were published between 2001 and 2019, and among them, seven studies^6,23,24,26–29^ were conducted between 1999 and 2014. The included studies were conducted in Angola^22^, Kenya^23^, Uganda^24,26^, Tanzania and Uganda^25^, Southern Sudan^27^, Sudan^28^, and Democratic Republic of Congo^29^, whereas one study was conducted in several countries (Angola, Central African Republic, Côte d’Ivoire, Democratic Republic of Congo, Equatorial Guinea, Republic of Congo, and Southern Sudan)^6^ (Fig. 2). Two studies^22,25^ were clinical trials, two^23,24^ were retrospective studies, three^6,28,29^ were cohort studies, one^27^ was a cross-sectional study, and one^26^ was a case–control study. Four studies^23,24,26,27^ enrolled patients with early- and late-stage HAT, whereas the other studies^6,22,25,28,29^ enrolled only patients with late-stage HAT. All included studies used the microscopic method to examine malaria parasites. Four studies^6,22,27,28^ used the agglutination test for trypanosomiasis as a serological screening test followed by microscopy (direct in blood, lymph aspirate, or cerebrospinal fluid or trough concentration by hematocrit centrifugation technique), whereas other studies used microscopy^23,24,29^ or microscopy/molecular methods^25,26^ to examine *Trypanosoma* spp. (Table 1). Eight studies^22–29^ reported the number of *Plasmodium* spp. infections among patients with HAT, whereas only one study reported that malaria trophozoites were detected in half of the patients tested for malaria in the initial blood smear^6^. Among 1555 patients with HAT in eight studies^22–29^, 707 patients were infected by *Plasmodium* spp.. Among these patients, four studies^6,22,27,28^ reported cases of *Plasmodium* spp. infection (534 cases) among patients with gambiense HAT (1181 cases), and the other studies^23–26^ reported *Plasmodium* spp. infection (173 cases) among patients with rhodesiense HAT (374 cases).

**Table 1:** Characteristics of the included studies.

Author (reference)	Study site	Year of study	Study design	Participants	Age	Male (%)	Number of co-infected patients	Number of patients with HAT	Type of HAT	Impact of co-infection	Test for malaria	Test for HATT
Blum et al., 2001^21^	Angola	NS	Clinical trial	588 patients with late-stage HAT	NS	NS	14	16	Gambiense HAT	Malaria might influence the risk of encephalopathy syndrome	Microscopy	CATT as a serological screening test followed by microscopy (direct using blood, lymph aspirate, or CSF or trough concentration by HCT capillary centrifugation)
Blum et al., 2007	Democratic Republic of Congo	2004–2005	Cohort study	60 patients with late-stage HAT and 60 healthy controls	HAT: 34.5 ± 11.8 (matched controls)	HAT: 48.3%	60	60	Gambiense HAT	HAT patients co-infected with malaria had a significantly longer corrected QT time	Microscopy	Microscopy (direct using blood, lymph aspirate, or CSF)
Blum et al., 2006^6^	Angola, Central African Republic, Côte d’Ivoire, Democratic Republic of Congo, Equatorial Guinea, Republic of Congo, and Southern Sudan	1999–2002	Cohort study	2541 patients with late-stage HAT	Mean 9.1 ± 15.1	49.5	Malaria trophozoites were detected in half of the patients tested for malaria in the initial blood smear	2541	Gambiense HAT	NS	Microscopy	CATT as a serological screening test followed by microscopy (direct using blood, lymph aspirate, or CSF or trough concentration by HCT capillary centrifugation
Kagira et al., 2011^22^	Kenya	2000–2009	Retrospective study	31 patients with early- or late-stage HAT	Mean 32.5 ± 2.3, range 14–57	61.3	31	31	Rhodesiense HAT	Malaria infection might increase the risk of drug toxicity	Microscopy	Microscopy (by HCT capillary centrifugation)
Kato et al., 2015^23^	Uganda	2005–2012	Retrospective study	258 patients with early- or late-stage HAT	Mean 28.6	48.1	70	242	Rhodesiense HAT	Malaria did not significantly affect HAT clinical presentation and case fatality rates, co-infected individuals had longer hospital admissions coupled with higher treatment costs	Microscopy	Microscopy (direct using blood, lymph aspirate, or CSF)
Kuepfer et al., 2011^24^	Tanzania and Uganda	NS	Clinical trial	138 patients with late-stage HAT	Mean 35 ± 19, range 6–85	57.2	57	69	Rhodesiense HAT	Co-infections with malaria and HIV did not influence the clinical presentation nor treatment outcomes	Microscopy	Microscopy (direct using blood and CSF by HCT capillary centrifugation); in some cases, molecular methods can be used Molecular method
Maina et al., 2010^26^	Southern Sudan	2003	Cross-sectional study	50 patients with early- or late-stage HAT	Mean 24 (0.25–62)	46	15	50	Gambiense HAT	Malaria co-infection was more common in females. Malaria co-infection in patients aged 10–19 and 20–39 was 1:1, however, SS mono-infection was more common in patients aged 20–29 than 10–19. SS mono-infection demonstrated lower WBC reduction (67%) than malaria co-infection (16.5%)	Microscopy	CATT as a serological screening test followed by microscopy (direct using blood, lymph aspirate, or CSF or trough concentration by HCT capillary centrifugation
Nsubuga et al., 2019^25^	Uganda	2013–2014	Case–control study	32 patients with early- or late-stage HAT	Mean 28.8 ± 14.1	50%	15	32	Rhodesiense HAT	The TNF-α level was significantly elevated in co-infection over HAT or malaria mono-infections	Microscopy, nested PCR	Microscopy, HCT capillary centrifugation, molecular method
Priotto et al., 2008	Sudan	2001–2002	Cohort study	1055 patients with late-stage HAT	Median 22 (15–32)	56%	445	1055	Gambiense HAT	NS	Microscopy or rapid diagnostic test	CATT as a serological screening test followed by microscopy (direct using lymph node aspirate or blood, HCT capillary centrifugation, or quantitative buffy coat techniques

*NS* not specified, *HAT* human African trypanosomiasis, *CATT* card agglutination, test for trypanosomiasis, *HCT* hematocrit, *CSF* cerebrospinal fluid.

### Risk of bias

The risk of bias among the included studies was assessed using the checklist for analytical cross-sectional studies developed by the JBI. A score of 7 points was given for five studies^23,24,27–29^, which were considered to have a low risk of bias, whereas four studies^6,22,25,26^ were given scores of 5–6 points and categorized as having a moderate risk of bias (Table S2).

### Prevalence of *Plasmodium* spp. infection among patients with HAT

**Figure 3:**
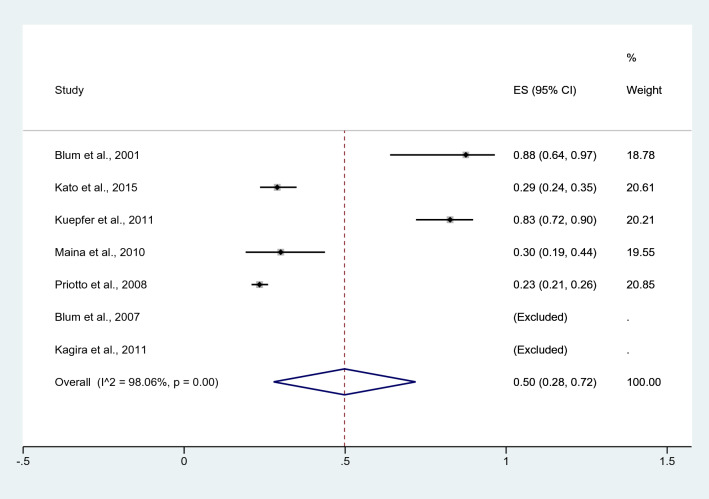
Pooled prevalence of malaria in human African trypanosomiasis. *ES* effect size, *CI* confidence interval; black diamond symbol, point estimate, Dashed line: pooled prevalence of malaria in human African trypanosomiasis; I^2^, level of heterogeneity; p = 0.00 or less than 0.05, significant heterogeneity.

The prevalence of *Plasmodium* spp. infection (692 cases) among patients with HAT was estimated using the data (n/N) of seven non-case–control studies that enrolled a total of 1523 patients with HAT^22–25,27–29^. Among the individual studies, Two studies by Kagira et al.^23^ conducted in Kenya and by Blum et al.^29^ conducted in the Democratic Republic of Congo reported *Plasmodium* spp. infection rates of 100% among patients with HAT. Meanwhile, two studies^22,25^ conducted in Angola, Tanzania and Uganda showed the high prevalence of *Plasmodium* spp. infection among patients with HAT at 88% and 83%. Lower rates were recorded in other studies (18–30%)^24,27,28^. Overall, the pooled prevalence of *Plasmodium* spp. infection among patients with HAT was 50% (95% confidence interval [CI] = 28–72%, *I*^*2*^ = 98.1%, seven studies; Fig. 3).

**Figure 4:**
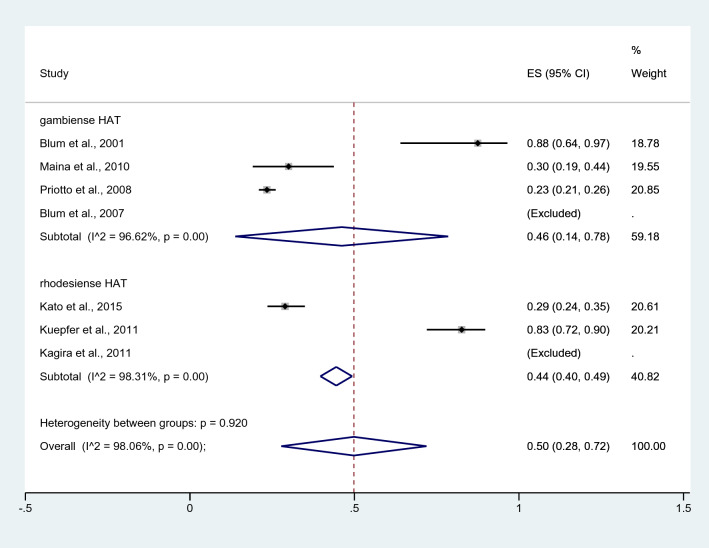
Subgroup analysis of the pooled prevalence of malaria in human African trypanosomiasis by HAT type. *ES* effect size, *CI* confidence interval; black diamond symbol, point estimate, Dashed line: pooled prevalence of malaria in human African trypanosomiasis; I^2^, level of heterogeneity; p = 0.00 or less than 0.05, significant heterogeneity.

Subgroup analysis by HAT type revealed that the pooled prevalence of *Plasmodium* spp. infection among patients with gambiense HAT was 46% (95% CI = 14–78%, *I*^2^ = 96.62%, four studies), whereas that among patients with rhodesiense HAT was 44% (95% CI = 40–49%, *I*^2^ = 98.3%, three studies, Fig. 4). The study by Kagira et al.^23^ reported a prevalence of *Plasmodium* spp. infection of 100% among patients with rhodesiense HAT. Meanwhile, the study by Kagira et al. Blum et al.^29^ reported a prevalence of *Plasmodium* spp. infection of 100% among patients with gambiense HAT.

### Effect of *Plasmodium* spp. infection in HAT

Seven studies^22–27,29^ determined the effect of *Plasmodium* spp. infection in HAT. Blum et al.^22^ suggested that malaria might influence the risk of encephalopathy syndrome, as 14 of 16 patients with HAT and encephalopathy syndrome of the coma type were infected with malaria (87.5%) in Angola. Blum et al.^29^ demonstrated that patients with HAT who were co-infected with *Plasmodium* spp. had a significantly longer corrected QT time (QTc). Kagira et al.^23^ suggested that malaria might increase the risk of drug toxicity; however, the effect of *Plasmodium* spp. in HAT in Kenya was not investigated in their study. The study by Kato et al.^24^ conducted a retrospective study in Uganda and concluded that malaria did not significantly affect the clinical presentation or death rates of HAT. However, longer hospital stays and higher treatment costs were recorded among co-infected individuals. Kuepfer et al.^25^ conducted a clinical trial of patients with HAT in Tanzania and Uganda and also demonstrated that *Plasmodium* spp. did not influence the clinical presentation or treatment outcomes of HAT. Maina et al.^27^ conducted the cross-sectional study of HAT in Sudan demonstrating that *Plasmodium* spp. infection in HAT was more common in female patients. In addition, co-infection was observed in patients aged 10–19 and 20–39, whereas *Trypanosoma* spp. mono-infection was more common in patients aged 20–29. Meanwhile, *Trypanosoma* spp. mono-infection was associated with greater total leukocyte reduction (67%) than malaria co-infection (16.5%)^27^. Nsubuga et al.^26^ conducted a case–control study of HAT in Uganda that assessed TNF-α levels and found that *Plasmodium* spp. infection was linked to higher TNF-α levels in patients with HAT than observed in patients with HAT or malaria mono-infections.

## Discussion

Testing for concomitant malaria infection in patients with HAT is not mandatory and is only conducted according to the decisions of the treating personnel^6^. In cases of malaria co-infection, antimalarial treatment is generally given as an ancillary drug before initiating HAT treatment to prevent encephalopathy^30–32^. The meta-analysis revealed that the high prevalence of malaria in HAT was high (50%). Nevertheless, the pooled prevalence appeared to be heterogeneous among the included studies. Subgroup analysis by HAT type indicated that the pooled prevalence of *Plasmodium* spp. infection was similar between patients with gambiense HAT and rhodesiense HAT (46% and 44%), and high heterogeneity was observed in the prevalence. Several reasons might explain the heterogeneity regarding prevalence among the included studies. The differences in the investigated participants might be the source of heterogeneity. For example, the study by Blum et al.^22^ examined *Plasmodium* spp. infection in patients with HAT and encephalopathy alone, whereas Kuepfer et al.^25^ examined *Plasmodium* spp. infection in patients with HAT and various types of complications. The participants in both studies had high rates of *Plasmodium* spp. infection. In addition to differences among the participants, the differences in the study design might be another cause of heterogeneity in the pooled prevalence. Two clinical trials^22,25^ included in the meta-analysis recorded a high prevalence of co-infection (83–88%). Blum et al.^22^ examined the blood slides of patients with HAT for malaria in the case of a reaction concurrent with fever, as malaria was suspected to increase the risk of encephalopathy in their participants; hence, the prevalence of malaria was high. Kuepfer et al.^25^ assessed malaria per a standard protocol to test co-infection with malaria at baseline and determine whether malaria influenced the clinical presentation or treatment outcomes in a Tanzanian study population; hence, the prevalence of malaria was high. The difference in the prevalence of co-infection might be explained by the difference in the study sites investigated. Blum et al.^22^ conducted their study in Angola, whereas the study by Maina et al.^27^ was performed in Sudan. The most recent WHO reports illustrated that the malaria burden share was higher in Angola (3%) than in Sudan (1%)^9^. Among studies conducted in East Africa, where *T.b. rhodesiense* is predominant, the study by Kagira et al.,^23^ which was conducted in Kenya, recorded a higher prevalence of co-infection (100%) than the studies by Kuepfer et al.,^25^ which was conducted in Tanzania and Uganda (83%), Kato et al.,^24^ which was conducted in Uganda (29%), and Nsubuga et al.,^26^ which was conducted Uganda (47%). A possible explanation of the high prevalence of *Plasmodium* spp. infection among patients with HAT (35–71%) was that it was mainly linked with the high prevalence of malaria in the general population of Africa, where malaria is highly endemic^8^.

The occurrence of *Plasmodium* spp. infection among patients with HAT suggested that these patients were susceptible to other infections, which might influence the pathogenesis and prognosis of the disease. As HAT is a chronic disease that leads to long-term immunosuppression^27,33^, which could increase the possibility of *Plasmodium* spp. infection in patients with HAT. Nevertheless, HAT is usually a chronic disease, but rhodesiense HAT often causes a more acute and rapidly progressive disease than gambiense HAT^34^. The role of immune responses in co-infected patients was demonstrated by Nsubuga et al.^26^, who revealed that co-infection by *P. falciparum* increased TNF-α levels in patients with HAT. Previous studies revealed associations of TNF-α with the progression and severity of HAT^35,36^. The synergistic effect of TNF-α on disease progression was related to increased IFN-γ levels^37,38^. The possibility of *Plasmodium* spp. infection among patients with HAT was probably high in previous studies^6,22,25,28^ that enrolled only patients with late-stage HAT. Although the study by Blum et al.^6^ that enrolled patients with late-stage HAT did not report the number of cases of *Plasmodium* spp. infection among their participants, a malaria prevalence of 50% among patients with HAT was recorded in several endemic countries including Angola, Central African Republic, Côte d’Ivoire, Democratic Republic of Congo, Equatorial Guinea, Republic of Congo, and Southern Sudan.

Testing for concomitant malaria infection in patients with HAT was not mandatory, and it was only performed according to the discretion of the treating personnel^6^. In cases of malaria co-infection, antimalarial treatment is generally given as an ancillary drug before initiating HAT treatment to prevent encephalopathy^30–32^. Because the examination of malaria parasites among patients with HAT was not mandatory, the possibility of *Plasmodium* spp. infection in HAT could not be discounted. The presence of fever in patients with HAT might support the presence of malaria parasites, and further examinations of malaria parasites are required. Fever was reported in half of the patients with HAT, resulting in a febrile reaction during HAT treatment^6^. If malaria parasites are present, then antimalarial treatment is generally given before initiating HAT treatment^31,32^. Antimalarial treatment was described by Blum et al.^22^ in which a full course of 1500 mg of chloroquine was given before the start of HAT treatment with melarsoprol.

Concerning the effect of *Plasmodium* spp. infection in HAT, a previous study found that the co-infected patients required multiple tests and experienced higher treatment costs because of the use of multiple drugs^23^. In addition, co-infected patients might have a poor prognosis due to prolonged hospital stay, receiving more drugs, and experiencing increased drug toxicity from melarsoprol treatment^23,24^. Kagira et al.^23^ observed that malaria could increase the toxicity of drug therapy. Suramin was injected in patients with early-stage rhodesiense HAT, whereas melarsoprol was used to treat patients with late-stage HAT^23^. Previous research demonstrated that the most serious side effect of melarsoprol treatment is post-treatment reactive encephalopathy characterized by increased mental deterioration, coma, and convulsions or death in some cases after treatment^39^. Blum et al.^29^ reported increased QTc in patients with HAT and concomitant malaria, suggesting the probability of melarsoprol-induced ventricular dysrhythmia in late-stage HAT. However, one study suggested that malaria and other co-infections did not significantly affect the clinical presentation and case fatality rates of HAT^24^. A similar observation was reported by Kuepfer et al.,^25^ who conducted a study in Tanzania and Uganda. They revealed that *Plasmodium* spp. infection did not influence the clinical presentation or treatment outcomes of HAT^25^. However, the outcome of this co-infection might be influenced by several factors such as the stage of infection and age and sex of the patient^40^.

The present study had several limitations. First, the number of studies that examined malaria and trypanosome co-infection was limited. Therefore, the differences in clinical characteristics, laboratory parameters, and treatment outcomes between co-infected patients and patients with malaria or HAT mono-infection could not be investigated. Second, there was high heterogeneity regarding the pooled prevalence of co-infection in the meta-analysis; therefore, the pooled prevalence should be carefully interpreted.

## Conclusion

Because of the high prevalence of malaria among patients with HAT and the endemic nature of malaria in Africa, malaria was present in some asymptomatic patients. Therefore, it is suggested to test every patient with HAT for malaria before initiating HAT treatment. If malaria is present, patients should receive antimalarial treatment before HAT treatment is initiated. Antimalarial treatment in patients with HAT might decrease the probability of poor clinical outcomes and case fatality rates.

## Supplementary Information

Supplementary Table S1.

Supplementary Table S2.
