# Supplementary material for: Prevalence and outcomes of malaria as co-infection among patients with human African trypanosomiasis: a systematic review and meta-analysis
Source: Sci Rep. 2021 Dec 10;11:23777. doi: 10.1038/s41598-021-03295-8 (PMC8664815; doi:10.1038/s41598-021-03295-8)
Supplement: Supplementary file 1 — Supplementary Table S1. [file 41598_2021_3295_MOESM1_ESM.docx]

**Prevalence and outcomes of malaria as co-infection among patients with Human African Trypanosomiasis: A systematic review and meta-analysis**

Kwuntida Uthaisar Kotepui^1^, Frederick Ramirez Masangkay^2^, Giovanni De Jesus Milanez^3^, Manas Kotepui^1*^

^1^ Medical Technology, School of Allied Health Sciences, Walailak University, Tha Sala, Nakhon Si Thammarat, Thailand

^2^ Department of Medical Technology, Institute of Arts and Sciences, Far Eastern University-Manila, Manila, Philippines

^3^ Department of Medical Technology, Faculty of Pharmacy, Royal and Pontifical University of Santo Tomas, Manila, Philippines

^*^Corresponding author

Manas Kotepui: [manas.ko@wu.ac.th](mailto:manas.ko@wu.ac.th), Tel.: +66954392469

Kwuntida Uthaisar Kotepui: [kwuntida.ut@wu.ac.th](mailto:kwuntida.ut@wu.ac.th)

Frederick Ramirez Masangkay; [frederick_masangkay2002@yahoo.com](mailto:frederick_masangkay2002@yahoo.com)

Giovanni De Jesus Milanez; gdmilanez@ust.edu.ph

**Table S1. Search term**

| **Databases** | **Search terms/Search strategy** | **Date** |
| --- | --- | --- |
| MEDLINE | (Malaria OR Plasmodium) AND (Trypanosomiases OR "Sleeping Sickness" OR "African Sleeping" OR Nagana OR Trypanosome OR Nannomona) AND (coinfection OR co-infection OR "Co infection" OR mixed OR concurrent OR Polymicrobial OR multiple OR dual)  Search results: 179 | 4 July 2021 |
| Scopus | (Malaria OR Plasmodium) AND (Trypanosomiases OR "Sleeping Sickness" OR "African Sleeping" OR Nagana OR Trypanosome OR Nannomona) AND (coinfection OR co-infection OR "Co infection" OR mixed OR concurrent OR Polymicrobial OR multiple OR dual)  Search option: Title, abstract, keywords  Search results: 54 | 4 July 2021 |
| Web of Science | (Malaria OR Plasmodium) AND (Trypanosomiases OR "Sleeping Sickness" OR "African Sleeping" OR Nagana OR Trypanosome OR Nannomona) AND (coinfection OR co-infection OR "Co infection" OR mixed OR concurrent OR Polymicrobial OR multiple OR dual)  Search option: All fields  Search results: 94 | 4 July 2021 |
