# Supplementary material for: Prevalence and outcomes of malaria as co-infection among patients with human African trypanosomiasis: a systematic review and meta-analysis
Source: Sci Rep. 2021 Dec 10;11:23777. doi: 10.1038/s41598-021-03295-8 (PMC8664815; doi:10.1038/s41598-021-03295-8)
Supplement: Supplementary file 2 — Supplementary Table S2. [file 41598_2021_3295_MOESM2_ESM.docx]

**Prevalence and outcomes of malaria as co-infection among patients with human African trypanosomiasis: A systematic review and meta-analysis**

Kwuntida Uthaisar Kotepui^1^, Frederick Ramirez Masangkay^2^, Giovanni De Jesus Milanez^3^, Manas Kotepui^1*^

^1^ Medical Technology, School of Allied Health Sciences, Walailak University, Tha Sala, Nakhon Si Thammarat, Thailand

^2^ Department of Medical Technology, Institute of Arts and Sciences, Far Eastern University-Manila, Manila, Philippines

^3^ Department of Medical Technology, Faculty of Pharmacy, Royal and Pontifical University of Santo Tomas, Manila, Philippines

^*^Corresponding author

Manas Kotepui: [manas.ko@wu.ac.th](mailto:manas.ko@wu.ac.th), Tel.: +66954392469

Kwuntida Uthaisar Kotepui: [kwuntida.ut@wu.ac.th](mailto:kwuntida.ut@wu.ac.th)

Frederick Ramirez Masangkay; [frederick_masangkay2002@yahoo.com](mailto:frederick_masangkay2002@yahoo.com)

Giovanni De Jesus Milanez; [gdmilanez@ust.edu.ph](mailto:gdmilanez@ust.edu.ph)

**Table S2.** Quality of the included studies

| No. | Authors | Eligibility criteria | Study subjects and the setting | Exposure measured in a valid and reliable way 'gold standard' | A specified diagnosis or definition | Confounding factors | Dealing with confounding factors | Outcomes measured in a valid and reliable way | Appropriate statistical analysis | Scores (8) | Risk of bias (low, moderate, high) |
| --- | --- | --- | --- | --- | --- | --- | --- | --- | --- | --- | --- |
| 1 | Blum et al., 2001 | Yes | No | Yes | Yes | No | NA | Yes | Yes | 6 | Moderate |
| 2 | Blum et al., 2006 | Yes | No | Yes | No | No | NA | Yes | Yes | 5 | Moderate |
| 3. | Blum et al., 2007 | Yes | Yes | Yes | Yes | Yes | NA | Yes | Yes | 7 | Low |
| 3 | Kagira et al., 2011 | Yes | Yes | Yes | Yes | No | NA | Yes | Yes | 7 | Low |
| 4 | Kato et al., 2015 | Yes | Yes | Yes | Yes | No | NA | Yes | Yes | 7 | Low |
| 5 | Kuepfer et al., 2011 | Yes | No | Yes | Yes | No | NA | Yes | Yes | 6 | Moderate |
| 6 | Maina et al., 2010 | Yes | Yes | Yes | Yes | No | NA | Yes | Yes | 7 | Low |
| 7 | Nsubuga et al., 2019 | Yes | No | Yes | Yes | No | NA | Yes | Yes | 6 | Moderate |
| 8 | Priotto et al., 2008 | Yes | Yes | Yes | Yes | Yes | NA | Yes | Yes | 7 | Low |

NA, Not Applicable
